# Shaping anatomical theatre’s teaching environment: Antonio Scarpa and the places of anatomy

**DOI:** 10.1007/s00276-026-03883-1

**Published:** 2026-05-11

**Authors:** Marcello Trucas, Emanuele Armocida, Valentina Cani, Michela Vincis, Denis Barry, Maria Carla Garbarino

**Affiliations:** 1 Discipline of Anatomy, School of Medicine, Trinity College Dublin, 152 - 160 Pearse St, Dublin, D02 R590 Ireland; 2 Department of Biomedical Sciences, Cittadella Universitaria, SS 554, km 4.5, 09042 Monserrato, Italy; 3 Department of Medicine and Surgery, University of Parma, Str. dell’Università, 12, 43121 Parma, Italy; 4 University Museum System, University of Pavia, S.da Nuova, 65, 27100 Pavia, Italy; 5 Department of Medical Sciences and Public Health, Unit of Anatomical Pathology, University of Cagliari, Cittadella Universitaria, 09042 Monserrato, Italy; 6 Department of Brain and Behavioral Sciences, University of Pavia, Policlinico San Matteo Viale Golgi 19, 27100 Pavia, Italy

**Keywords:** Anatomy, Dissection, Anatomical theatres, Anatomical education, Surgery, Antonio Scarpa

## Abstract

**Purpose:**

Antonio Scarpa (1752–1832) was an Italian anatomist who represented an important junction point between clinical and didactic anatomy and surgery. His discoveries and anatomical illustrations offer valuable insights that have contributed meaningfully to the development of modern medicine and anatomy. By combining his roles as physician, surgeon, and anatomist, he established himself as a respected figure among anatomists and medical specialists of his time. However, Scarpa’s contribution to the development of spaces dedicated to anatomical teaching remains relatively overlooked. This study aims to highlight his influence on the evolution of institutions and environments where anatomy was taught and practised.

**Methods:**

Through literature review and archival documents, we have obtained original data on the fact that Scarpa has always considered teaching, and its place as a fundamental component for the progress of anatomical sciences and not a marginal aspect.

**Results:**

We have analysed the history of the places where he studied, taught, and gave an indelible imprint on anatomical theatres, which we inherit today in modern dissecting theatres. From our study, it emerges that Scarpa’s ideas on how to disseminate anatomical knowledge were shaped by his experiences in Padua, Modena, and Pavia, places that played a meaningful role in refining his vision of anatomical education. At the same time, Scarpa himself contributed to transforming these institutions and their anatomical spaces, leaving a lasting mark on how anatomy was practised.

**Conclusions:**

The present work contributes to the historiography of anatomical education, offering new insights into the relationship between anatomical knowledge and its spatial contexts.

## Purpose

Antonio Scarpa (1752–1832) holds a place of honour in the history of anatomy and in the history of medicine in general; his authority was, in fact, recognised internationally [21]. He has been known for his many contributions in numerous fields. Anatomist, researcher, and academic lecturer, his input to the field of anatomy and neuroanatomy has found application in many surgical disciplines [11, 14]. Scarpa was among the first to underline the relationship between form and function in organs, particularly in the nervous system, influencing other great neuroanatomists who made important discoveries thanks to this approach [38].

In general surgery, he is most known for the fascia of Scarpa, and for what is now called the Scarpa triangle, an area he described for accessing the femoral artery, which is bordered by the sartorius muscle laterally, the adductor longus muscle medially, and the inguinal ligament superiorly [21].

Antonio Scarpa, after recognising the educational value of anatomical dissection in Padua’s theatre, went on to teach in Modena, where he helped design a theatre for his lessons. Later, in Pavia, he once again focused on building an anatomical theatre. Anatomical theatres built from the late 16th to the 19th century were unique in their architectural design, hosting significant medical cultural evolution. Today, the anatomical theatres in Padua, Modena, and Pavia still preserve their original structures and can be visited as part of museum tours [23].

The birth of modern anatomy is generally considered secondary to Mondino de Liuzzi’s reintroduction of the dissection of the human cadaver, when, having overcome the taboo that considered the autopsy of the corpse as a violation of the sacredness of the human body, public anatomy lessons spread in European universities [24]. The affirmation of Humanism led to significant advancements in anatomical studies, with Vesalius’s work in the 16th century. During the Enlightenment, medicine moved away from dogma towards experimental methods. The evolution of modern scientific approaches is closely tied to the renewal of humanistic perspectives, resulting in cultural and social progress. Science, religion, and society open a debate that effectively puts man at the centre of their reflections with an anthropocentrism characteristic of a humanistic approach, ideally meeting and finding themselves in anatomical theatres, thanks also to anatomists who also fought against superstitions in science [37].

Architecturally, the first anatomical theatres are presented as works of a celebratory nature, intended to bear witness to the establishment’s attention to cultural development [23]. However, their structures were functional for demonstration-based teaching needs and linked to the intention of spreading knowledge even outside the narrow circle of scholars. Historically, to achieve these goals, few anatomists have tried to play a key role, participating in the design phase to adapt architectural spaces to their needs, but it could depend on individual circumstances. This work explores how Antonio Scarpa played a crucial role in linking anatomical theatres, anatomy education, surgeons, and humanists.

## Methods

We conducted a comprehensive historical review combining secondary literature with primary archival sources. Original documents were retrieved from the Historical Archives of the University of Pavia, where Scarpa held his most influential academic role. This choice reflects his mature period, during which he exerted significant political and scientific influence on decisions regarding anatomical facilities. The archival material included manuscripts, correspondence, and architectural plans, which were critically examined to reconstruct Scarpa’s vision of anatomical theatres. These sources were integrated with published historical studies to highlight how Scarpa consistently regarded teaching, and the architectural spaces dedicated to it, as a fundamental component for the advancement of anatomical sciences, rather than a marginal aspect.

## Results

The findings are presented in three chronological phases: Scarpa’s formative experience in Padua, his innovative initiatives in Modena, and his mature period in Pavia, during which archival evidence demonstrates his consolidated authority and decisive role in shaping anatomical facilities.

## Scarpa in Padua: the birth of a talent in a great school (and place)

**Fig. 1:**
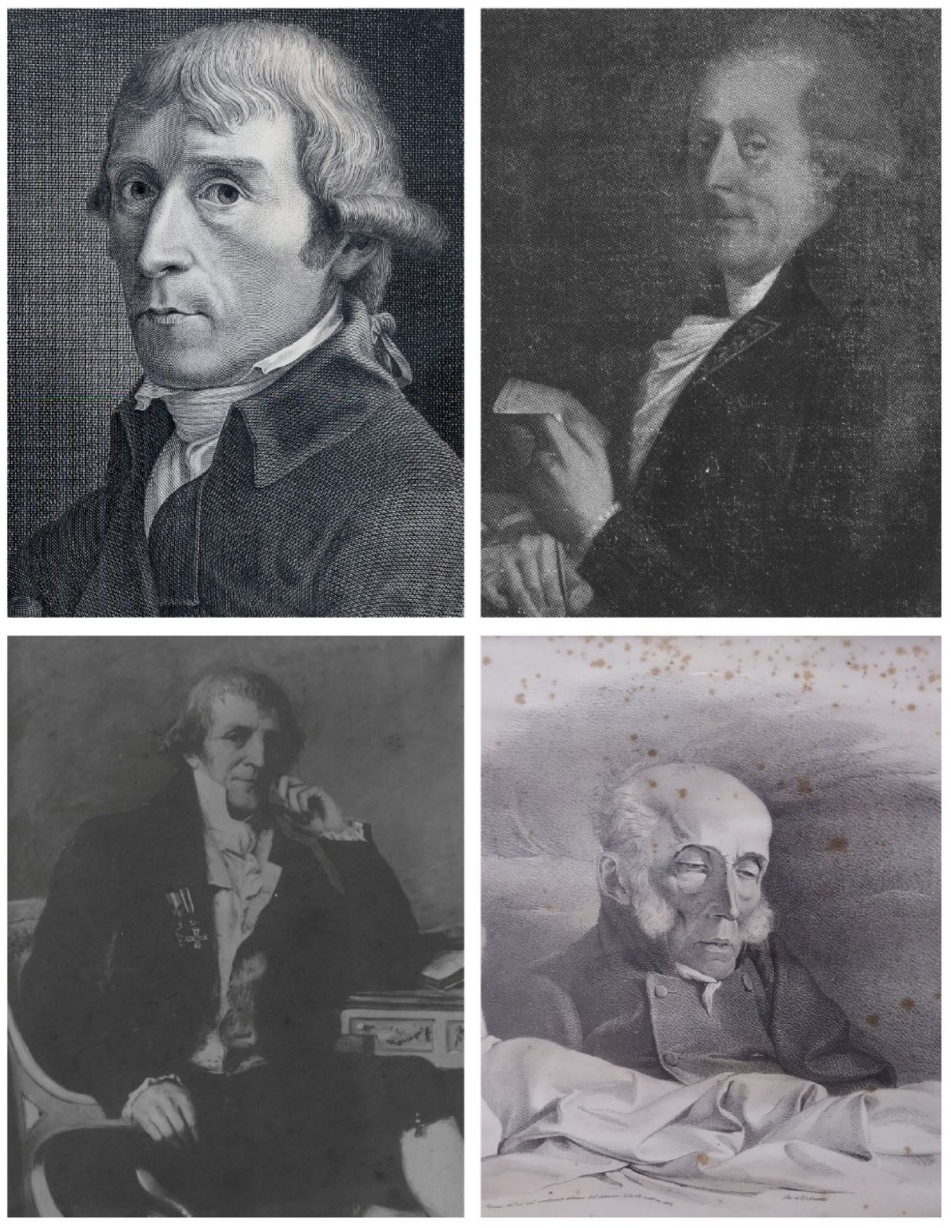
Four portraits of Antonio Scarpa. University of Pavia. Top left: Scarpa in 1801, a life drawing by G. Cattaneo and an engraving by F. Anderloni [32]; top right: Scarpa in 1806, an oil portrait by A. Appiani; bottom left: a posthumous portrait, oil on canvas by G. Bertini, 1894; bottom right: Scarpa, post-mortem portrait by Cesare Ferreri, 1832

Antonio Scarpa was born in Motta di Livenza, in the Veneto region of Italy, in 1752 (Fig. 1).

Son of a boatman, Scarpa was introduced to formal studies by his paternal uncle, who was a priest.

At the age of 14, Antonio entered the Medical School of the University of Padua, where he was mentored by the surgeon Girolamo Vandelli and by Giovanni Battista Morgagni, who is regarded as the father of anatomical pathology [16]. According to Scarpa himself, Morgagni quickly recognised his talent and appointed him as both assistant and personal secretary. This close mentorship allowed Scarpa to actively organise and participate in Morgagni’s lectures, marking the beginning of his academic journey. In 1770, at the age of 18, Scarpa graduated in philosophy and medicine [12, 21].

Scarpa was fortunate not only to have an excellent teacher like Morgagni, also considered the father of modern evidence-based medicine [40], but also to study anatomy in a place that had a primary role in the development of modern anatomy, experimental medicine, and demonstration teaching, as we will see below.

The concept of the anatomical theatre originated in Padua and was first described by Italian anatomist Alessandro Benedetti in his 1493 treatise *Historia corporis humani sive Anatomice*. These theatres were temporary wooden structures that were disassembled after use [23]. Benedetti, who obtained his degree in Padua in 1475, practised medicine on the island of Crete before teaching anatomy and surgery in Padua from 1490 [10]. He was also a humanist and author of many non-medical works. His *Historia* was the first work after Mondino de’ Liuzzi’s *Anothomia* to be entirely devoted to anatomy, serving as a descriptive compendium rather than a dissection manual. The *Historia* was widely published in Europe with several editions [27].

**Fig. 2:**
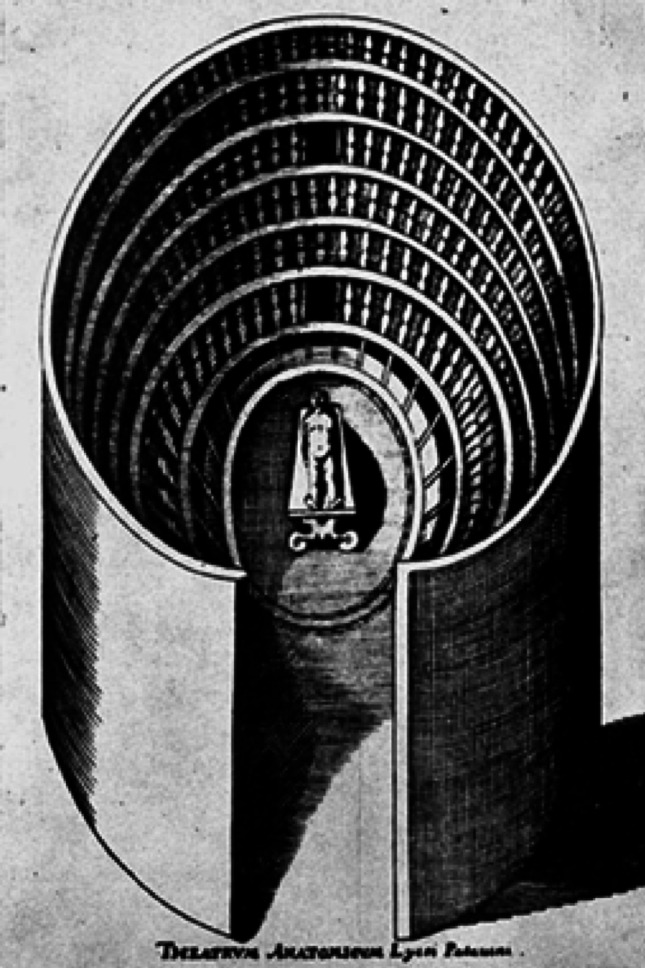
Plan of the anatomical theater of Padua. Gymnasium Patavinum, Jacobi Philippi Tomasini (1654)

The beginning of modern anatomy is marked by the publication of *De humani corporis fabrica* (in 1542–1543) by Andreas Vesalius (1514–1564). Vesalius, a lecturer at the University of Padua, revolutionised anatomical research by emphasising direct observation and dissection of cadavers. Before Vesalius, anatomical lessons involved three separate roles: the lector (reader), ostensor (pointer), and sector (dissector). Vesalius unified these roles, prioritising firsthand dissection experience over authoritative texts, correcting many errors in Galen’s works [5, 17]. This new approach established Padua as a leading secular scientific centre in Europe. After Vesalius died in 1564, the chair of surgery and anatomy at Padua was vacant until 1565, when Hieronymus Fabricius ab Aquapendente (1533–1619) was appointed. Fabricius, born in 1533 in Aquapendente, initially studied Greek, Latin, and philosophy at the University of Padua before studying medicine under Gabriele Falloppio (1523–1562). In 1594, faithful to Benedetti’s recommendations, Fabricius built the first permanent theatre ever designed for public anatomical dissections (Fig. 2), thus revolutionising the teaching of human anatomy. In this place, Scarpa studied. Used until 1872 and still occupying its original place in the ancient building of the University of Padua, the so-called Palazzo Bo, it certainly had the young Antonio Scarpa as a student [27].

The anatomical theatre of Padua was built by a complex interdisciplinary team coordinated by Fabricius d’Acquapendente, which involved Dario Varotari (1539–1596), painter and architect, and Paolo Sarpi (1552–1623), theologian and expert in optical matters [28, 29].

**Fig. 3:**
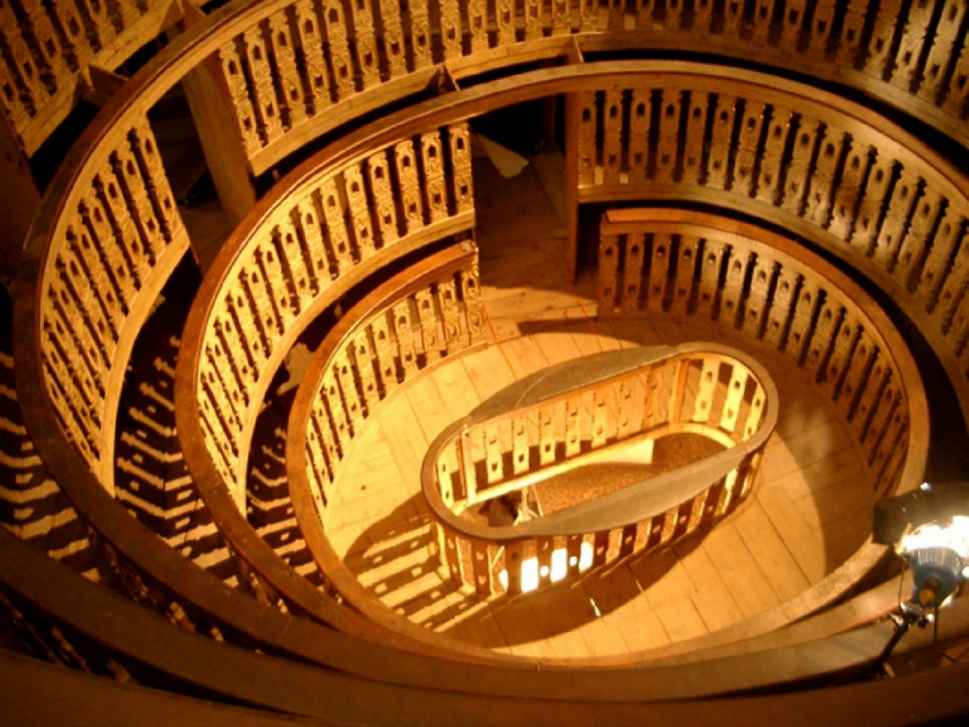
Anatomical theatre of Padua seen from above. The first four rounds of balustrades can be seen. Photo by Marco Bisello

The anatomical theatre of Padua is an original structure: a vaguely Dantesque funnel, maybe inspired by Galileo’s philosophy [19].

where the public was distributed over six overlapping groups (Fig. 3). Groups are not equipped with actual seats; the space between the balustrade and the base of the next group is 40 cm. The theatre materialises a real inverted visual cone, with the vertex coinciding with the centre of the anatomical table, on which the teacher teaches and demonstrates, enhancing experimental science [4].

The main constraints imposed on Fabricius d’Acquapendente in carrying out the project were due to the small size of the pre-existing masonry room. The choice of wood as a constructive element was dictated by the need to use light material and, above all, by the experimental nature of the intervention (to facilitate any variations or additions). It is interesting to note how the local workers had gained some experience in the prefabrication of ephemeral wooden structures, reasonably attributable to the continuous contact with the Venetian arsenals (many of the solutions adopted, both from a dimensional point of view and a technical-constructive point of view, refer to naval architecture) [18].

Initially, the desk where the corpse was placed and where the lesson took place was at the floor level of the room. The current wooden stalls, about two meters high above the ground, were arranged in 1845 by the then professor of anatomy, Francesco Cortese (1802–1883), who also had the windows opened to illuminate the Theatre with daylight [7].

Until 1844, the Theatre was artificially lit as the room didn’t have windows. The light was given by two candlesticks placed at the end of the anatomical table and by eight candles held by as many students seated on mobile benches. This method of lighting will probably remain rather unpleasant in the mind of Scarpa, who, as we will see later, will try in every way to have as much natural lighting as possible in his workplaces. The theatre is open to the public, but today it is only allowed to access the ground floor without being able to go up to the wooden levels in order to preserve them.

**Fig. 4:**
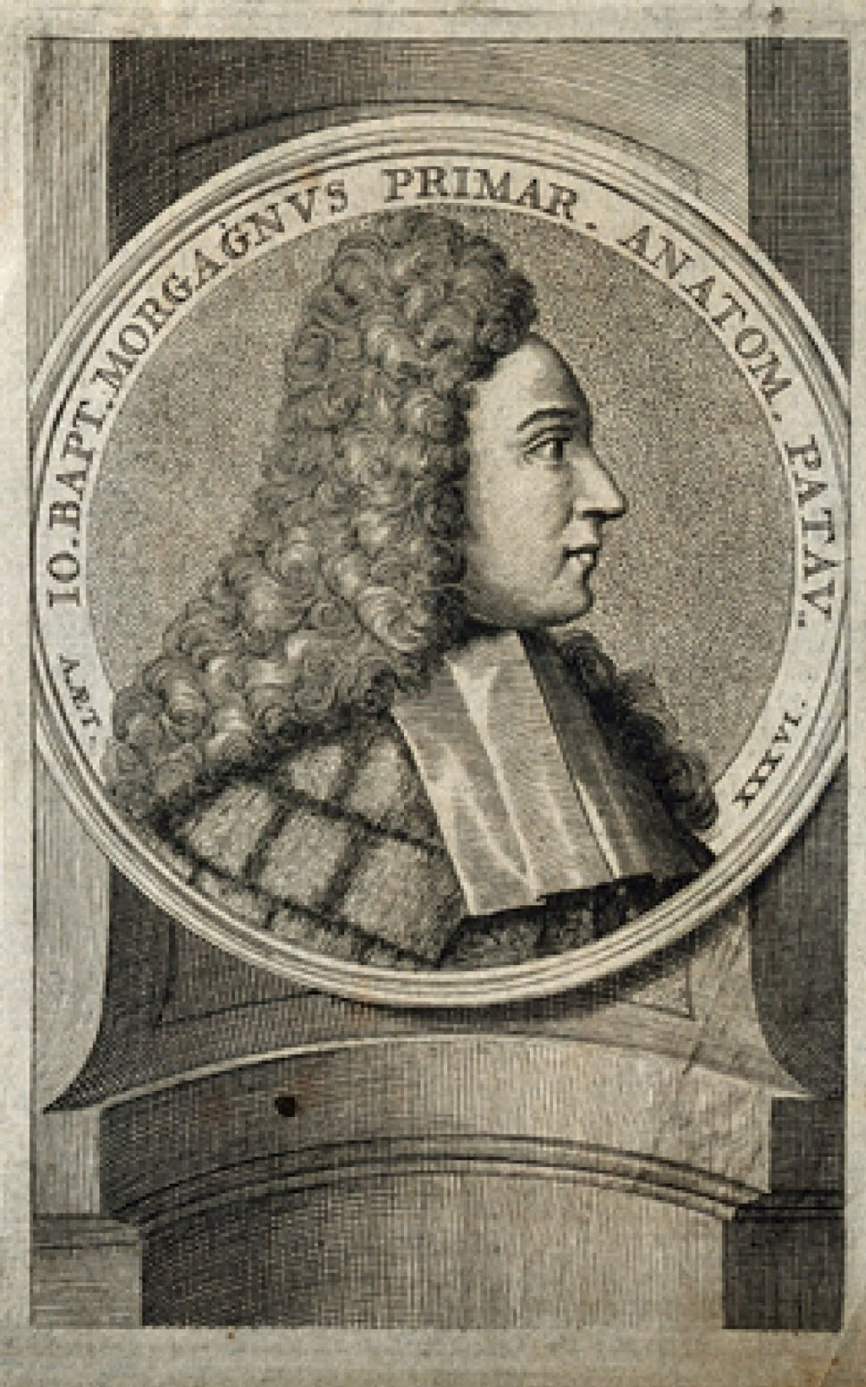
Giovanni Battista Morgagni. Line engraving. Wellcome Collection

The years of study in Padua were fundamental for the training of Scarpa, who, as well as with Morgagni (Fig. 4), collaborated assiduously with Luigi Calza (1737–1873), professor of obstetrics. The latter had been tasked with setting up a Cabinet of wax preparations for teaching obstetrics and had involved Scarpa in following the anatomical part. This interest in obstetrics would later develop during Scarpa’s stay in Modena. Similarly, studies on the internal ear would also have seen publication only after Scarpa’s move to Modena [34].

### Scarpa in Modena: realising an ambitious project

**Fig. 5:**
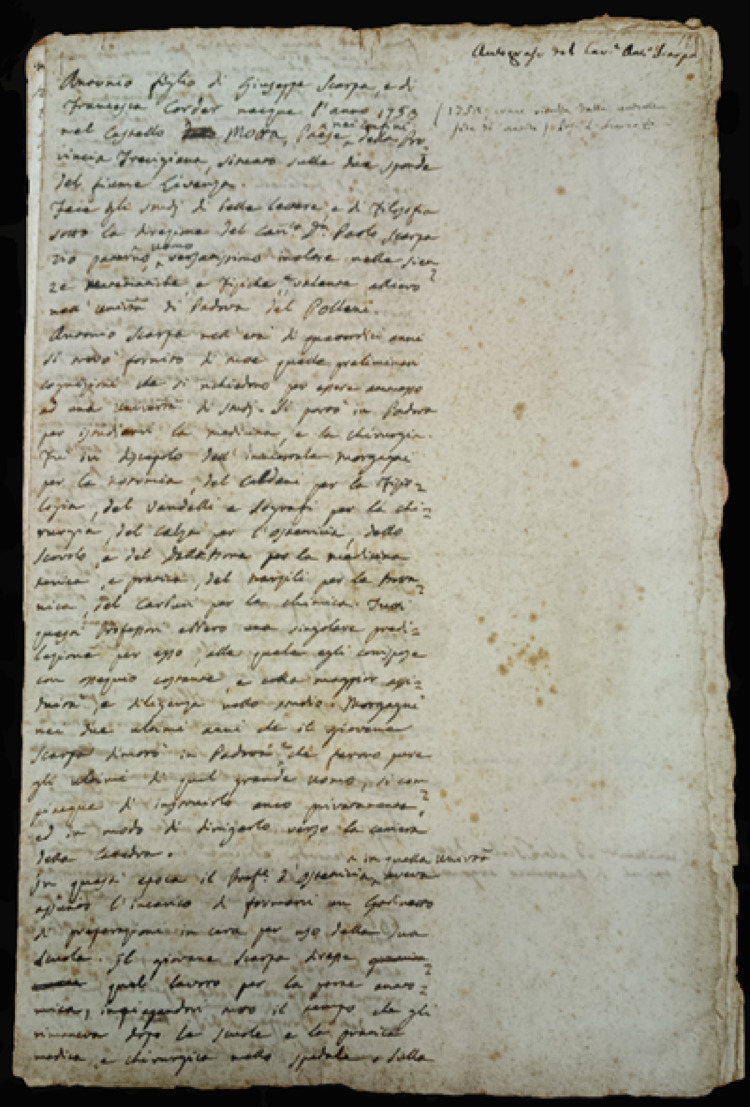
Scarpa’s handwritten *curriculum vitae*. Museum for the History of the University of Pavia

In 1772, at the age of 20, possibly on the recommendation of the surgeon Girolamo Vandelli (1699–1776)—another one of his professors at the University of Padua—Scarpa became a professor of anatomy and surgery at the University of Modena, where he would stay for a decade. From Padua, he brought with him the memory of Morgagni, who was always a model for him and compared to whom Scarpa—ambitious and proud—did not feel inferior. In his autobiographical curriculum (Fig. 5), unpublished manuscript preserved at the University History Museum of Pavia, he wrote, regarding the lessons in Modena: ‘The school was very crowded and also attended by very erudite men. they liked the style, order and clarity of the young Prof. (he is speaking of himself, n.d.r); and the traces of the great Master (Morgagni, n.d.r) he followed were recognised, nor was the copy of that noble model found ignoble’ [1].

Between 1775 and 1777, Scarpa also became the professor of obstetrics and gynaecology, as well as the chief surgeon of the Army Hospital in Modena [21]. It is worth remembering that after graduating, Scarpa practised surgery in Bologna as well, attending ‘[…] the main hospitals of the city’ [41]. It is conceivable that he visited the anatomical theatre of the Archiginnasio of Bologna, completely different from that of Padua: a room with a rectangular base that includes a largely fenestrated wall, seats for the students, the reader’s station and, near the table, the space of the dissector.

Scarpa, upon his arrival in Modena, was disappointed by the teaching means at his disposal. In fact, in the absence of an anatomical theatre, the dissections of cadavers were performed at the Civic Hospital of Sant’Agostino in a small room on the ground floor, and anatomy studies were reduced to theoretical lessons. Fortunately, a reform wanted by Francesco III d’Este (1698–1780) also provided for a renewal of the seats, including a stable anatomical theatre. He chose, in concert with Scarpa, to build the new building at the Sant’Agostino Hospital, in the area where the ancient church of S. Nicolò once stood. The hospital built between 1753 and 1758 by the will of the duke Francesco III himself, was managed by the Opera Pia Generale dei Poveri [6].

Ippolito Bagnesi, President of the Opera Pia Generale dei Poveri, commissioned Scarpa to personally take care of the internal structure of the anatomical theatre. This was finally an opportunity for Scarpa to express the basic needs of his teaching.

To convince Bagnesi of the architectural merits of the theatre built by Fabrizio Acquapendente, Scarpa had a wooden model sent to him by his former master Girolamo Vandelli, professor of surgical institutions at the University of Padua. Subsequently, two other models were made, one by the engineer Lodovico Bolognini and one based on a design by Lorenzo Toschi [6].

If the spaces, limited in an already existing room, constrained Fabricius d’Acquapendente’s projects, it was economic reasons that limited Scarpa’s ambitions in Modena. The presidents of Opera Pia chose Toschi’s model because it was less expensive. Work began in December 1773 and the theatre was officially inaugurated by Scarpa on 23 January 1775 [6].

From Toschi’s original drawings, initially, the actual anatomical theatre was housed in an octagonal masonry tower. Inside, the structure consisted of a wooden funnel with comfortable seats for students. It was a complete amphitheatre, with an elongated ellipse, but less high and less narrow than the one built in Padua based on a design by Acquapendente, with wider bleachers and equipped, at least in part, with benches. The arena is made up of 5 tiers of seats, the first four oval shapes, while the fifth reproduces the octagonal shape of the building.

The theatre was considered an artistic monument and had certificates of esteem that magnified the excellence of the work even outside Italy [9].

**Fig. 6:**
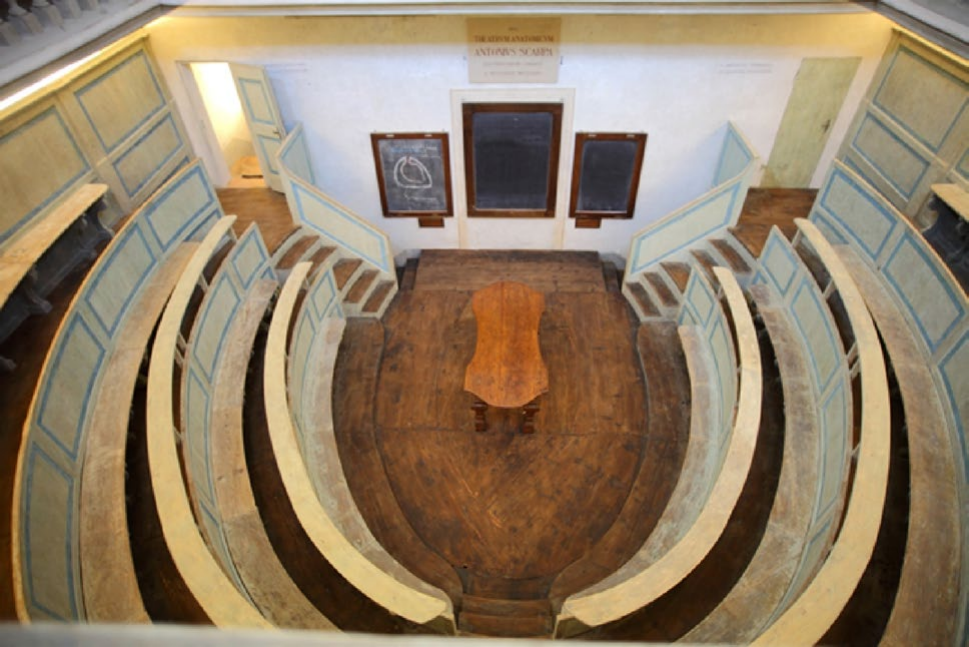
Anatomical theatre of Modena. Photo by Arch. Giovanni Coppini (Direzione Tecnica UniMoRe)

During Scarpa’s stay, the rooms adjacent to the theatre were set up to preserve the parts of the human body that were considered most interesting during dissection for educational and research purposes. The need to adequately accommodate the growing number of anatomical preparations prompted Archduke Francesco IV of Austria Este (1779–1846), in 1815, to have a new floor built above the anatomical theatre to create an anatomical museum. To give access to the upper floor, it was necessary to build a staircase, and the space was obtained by sacrificing a part of the area of the octagon of the theatre (Fig. 6), the one on the northern side facing the portico; in this way, the theatre took on an irregularly hexagonal plan, which it retains to this day. The theatre is open to the public and is included in the cultural visits of the former Sant’Agostino Hospital.

During his stay in Modena, Scarpa began to take care of the growth of an anatomical collection, an activity that he would later develop in Pavia as director of the Cabinet and Anatomical Museum of the University. In these years, moreover, ambitious and determined to increase his knowledge, Scarpa asked and was allowed to make educational trips (to France, England, and the Netherlands) that put him in contact with important European anatomists. On this occasion, Scarpa had a meeting that brought him to Pavia.

### Scarpa in Pavia: the pursuit of magnificence

In 1783, Scarpa was called to teach in Pavia by the Royals of Vienna at the suggestion of Giovanni Alessandro Brambilla (1728–1800), whom Scarpa had met during his stay in Paris. The latter was a very influential surgeon at the Austrian court, where he worked to ensure that army surgeons had better training and prestige comparable to that of doctors [13, 22]. In the past, army surgeons were often illiterate or poorly educated and, not knowing Latin, did not have access to the study of classical medical texts, including those on anatomy.

**Fig. 7:**
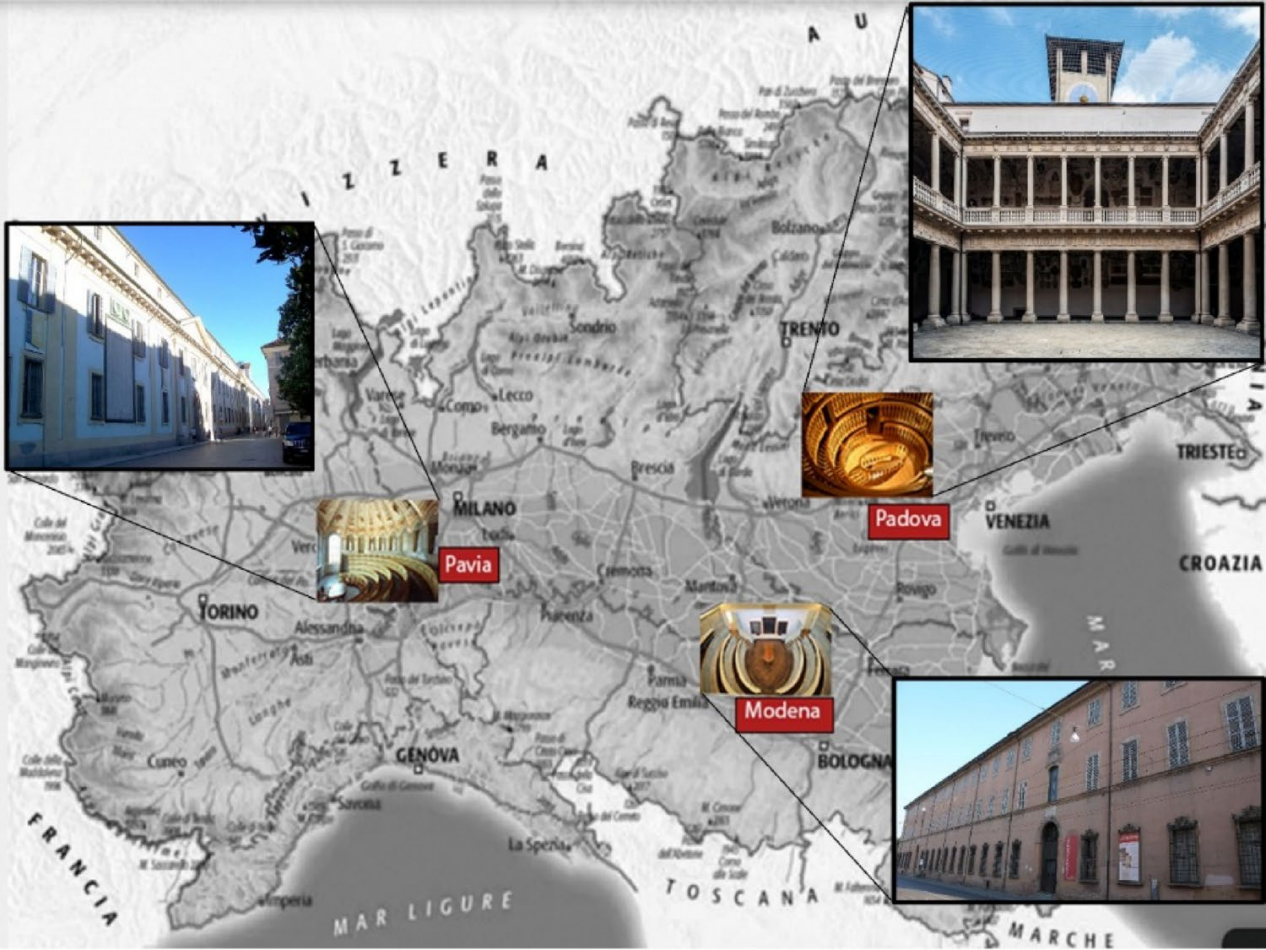
The map of the north of Italy indicating the location of the three anatomical theatres of Padua, Modena, and Pavia and their related external buildings

Brambilla strongly desired that the famous Scarpa pass from Modena to Pavia, to give renewed prestige to the chairs of anatomy and surgery and the entire university [25]. In the previous years, Maria Theresa of Austria had initiated a renewal of the University of Pavia, which was being equipped with important aids for teaching and research (cabinets, museums, libraries, places of study). Her son and successor, Joseph II, was continuing his mother’s policy (Fig. 7).

In 1783, Scarpa took up the chair of Anatomy and Surgical Institutions at the University of Pavia. He also directed the Surgical clinic from the year of its foundation, 1787, and held the chair of Clinical surgery. He was furthermore director of the Anatomical Museum, which, under his guidance, enriched with natural specimens and splendid wax models, became renowned throughout Europe.

In 1784, he undertook another educational journey, this time to Vienna, to visit the new scientific facilities that had been established in the Habsburg capital. The journey continued in the German states, touching Dresden, Berlin, Hannover, Göttingen, Augsburg, and Munich. In 1788, he was appointed, together with Johann Peter Frank (1745–1821), to lead the Medical-Surgical Directorate, transferred from Milan to Pavia and charged with overseeing the health professions and public hygiene.

Scarpa gained a position of great power at the University of Pavia, where he served several times as rector, as dean of the Faculty of Medicine, and as director of medical studies, leading the University during the dramatic political changes that affected Pavia between the late eighteenth century and the first thirty years of the nineteenth century. His prestige protected him. Being pro-Austrian, Scarpa asked to be placed on retirement when the French took control of Pavia. Napoleon himself, however, considered Scarpa as an indispensable prestige of the University of Pavia, and asked him to return to service, an honour that Scarpa was compelled to accept, perhaps with some measure of dissatisfaction [30].

A dark and perhaps unfair portrait of Scarpa has come down to us through some stories passed down in the medical faculty: his strong personality made him severe, little inclined toward indulgence, perhaps even cynical. He was miserly, and few people enjoyed his esteem, even fewer his affection. This dark portrayal, however, should perhaps be reconsidered in light of his generous commitment to research and teaching, and his ability to “protect” the University of Pavia and its students during the turbulent years between French rule and the Austrian Restoration.

When Scarpa arrived, the University of Pavia was equipped with a permanent anatomical wooden theatre, octagonal with a table at the centre and benches all around. It was accessed via stairs placed at the room’s four corners. It was made in 1671, probably on a project by the architect Giovanni Ambrogio Pessina [20].

A biographer of Scarpa wrote: ‘In giving principle to the anatomical demonstrations, Scarpa found in Pavia the same unpleasant conditions, which he found in Modena: lacking a skilled prosector and suitable place for such purpose. Therefore, the anatomical expositions had been made up to them in a kind of obscure catacomb lit by torches and even cramped to the great influx of national and foreign audiences’ [30].

Francesco Sartirana (1719–1790), Royal Councillor of the University, was asked to prepare a new project following the indications and necessities of Professor Scarpa. In the early years of the Pavia period, Antonio Scarpa began to establish himself as an international authority, and thanks to the important role held by the University of Pavia in the European scientific field [3], had the opportunity to entertain direct relationships with the various institutes of the continent. He visited the Institutes and Anatomical Museums of Vienna, Bohemia, Saxony, Prussia, Brunswichese, Hannover, Bavaria, Tyrol, Prague, Dresden, Leipzig, Berlin, Helmstadt, Göttingen, Paris, London [30].

In his correspondence, we found that Scarpa wrote about the intention to also have in Pavia an amphitheatre with a circular and very enlightened structure [35].

The structure designed by Sartirana had a perfectly circular shape, with an octagonal perimeter balustrade (like the pre-existing ancient one and similar to the original morphology of the one he inaugurated in Modena). It had to be very bright thanks to a large window at the top of the vault and five large windows on many sides. The benches would have been arranged on four levels plus a superior gallery, all accompanied by wooden balustrades.

The drawing was submitted to Leopoldo Pollack (1751–1806), an architect of Viennese origins and a pupil of Giuseppe Piermarini (1734–1808). He found it functional and satisfying despite the limited space available, and presented only a slight change, keeping the whole plan unchanged. For reasons of structural calculation, he was forced to reduce the diameter of the window at the top of the vault and proposed to make some lunette windows instead of the big windows at the base of the dome. These would have been useless in the public’s presence and would not allow the light’s passage.

However, this drawing was not realised because it did not appeal to either Emperor Joseph II or Chancellor Wenzel Anton von Kaunitz-Rietberg (1711–1794) [2, 20]. Despite the authority achieved by Scarpa in the Pavia period, also on this occasion, scientific reasons gave way to political will. In any case, it is important to underline that once again Scarpa dreamed of a circular structure, like the one in which he had studied in Padua, which led students to a direct observation of the dissection.

The Emperor preferred the semicircular plan, stating that ‘its entirely circular form does not seem to me the most appropriate*’* [8]. At that point, after a conference between Pollack and Scarpa to revise the project, his Majesty’s approval arrived following the report of the Court and State Chancellor, Giuseppe Firmian (1716–1782).

Regarding the decorations and the artistic aspects, he was certainly satisfied. Other features, such as the circularity of the cavea, the huge window at the top, the numerous windows around, and the gallery, were not made as Scarpa wished.

Scarpa’s correspondence gives us a direct testimony of the fact that he managed, despite various compromises, to create a theatre that made him fully satisfied and proud [35]. The comfort and beauty of the facility were also teaching tools to him. In the manuscript, preserved in the Museum for the History of the University, in which Scarpa lists the stages of his academic and scientific career, Scarpa declared that in 1785, in place of the ‘old narrow and dark anatomical theatre’, another one was built, ‘spacious, clear and magnificent’ [1]. In the same writing, he also underlined how his anatomical cabinet was well enough supplied with preparations to allow him to hold a complete anatomy course, even, it must be understood, when there were no bodies available to be subjected to autopsies in the anatomical theatre.

Brightness (‘clear’) was probably one of the characteristics that was most dear to Scarpa, as inferred from the fact that the designers strove to bring as much light as possible to the area of ​​the table for demonstrations [26]. Also, the five windows all around the seats testify to the great need for light that characterised his method (until then, in many theatres, it was necessary to light with torches and candles). This anticipated centuries the need for powerful surgical lamps used today in modern dissection rooms.

**Fig. 8:**
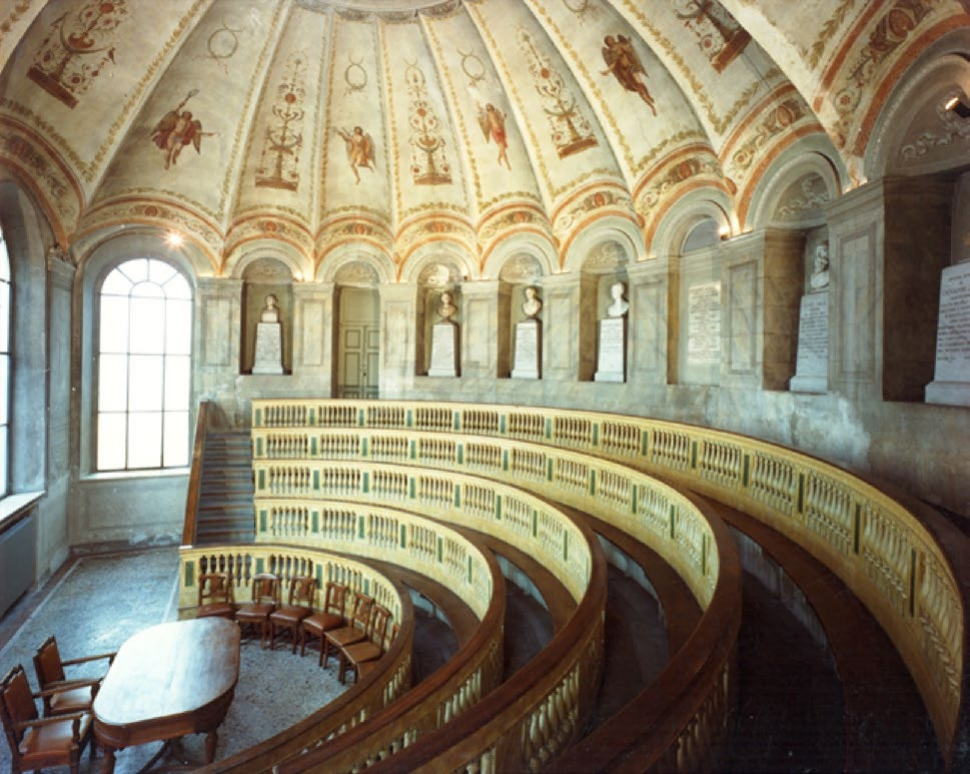
Anatomical Theatre of the University of Pavia, now the Aula Scarpa. University of Pavia

The anatomical theatre (Fig. 8) was inaugurated in October 1785, with works not yet completed, with a solemn inaugural oration by Prof. Scarpa himself [33]. In this oration, Scarpa, after a historical excursus on anatomical studies, exhorted the students not to fail in the example given by the great anatomists who had preceded them. His words urge the audience to maintain a deep connection with the humanities in medicine, urging students to cultivate the connection of the sciences with the art and history of their discipline. The work was completed in 1786, one year after the solemn inauguration [39].

**Fig. 9:**
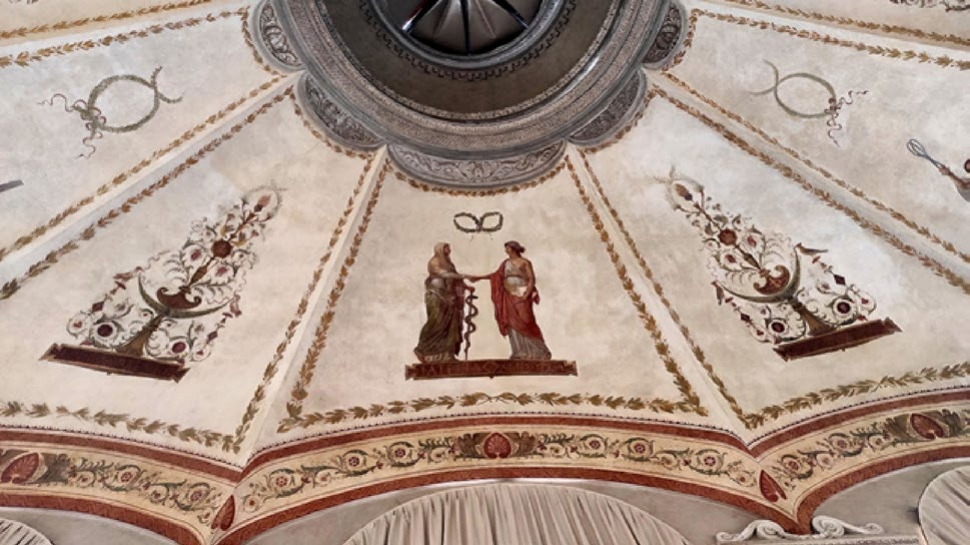
Medicine and Surgery Shaking Hands, fresco (c. 1819). University of Pavia, vault of the Aula Scarpa

The original decoration of Scarpa’s anatomical theatre has been lost. In 1819, a new ceiling was created, which probably received the approval of Scarpa, now elderly but still active in university life. The decorative apparatus of the room is a celebration of the rediscovered eighteenth-century ‘alliance’ between medicine and surgery. In the eighteenth century, the traditional distance between medicine and surgery had finally shortened, and figures such as Scarpa, an anatomist, physician and surgeon, had represented its synthesis. In the central part of the ceiling, we can admire two allegorical female figures holding hands: the elderly medicine, leaning on the rod of Asclepius, and the young surgery holding a scalpel in one hand. (Fig. 9) [20, 36].

Further down, behind the professor, were represented iconic figures of anatomical studies (Bartolomeo Eustachi, Gabriele Falloppio, Gaspare Aselli, and Giambattista Morgagni).

Even today, it is possible to visit the Theatre, used for the ceremonies of the University of Pavia.

The memorial plaque, placed in 1950, on the occasion of the restoration, testifies: ‘Anatomical Theatre, erected and adorned in 1785 on the advice and authority of Antonio Scarpa from Friuli […]’.

### Anatomy and art in Scarpa’s work

Just as Scarpa’s scientific and academic career took its first steps in Padua, the great tradition of anatomical illustration initiated by Vesalius’s *De humani corporis fabrica* was also destined to develop in his work. Scarpa possessed a great artistic talent and personally drew the images from which the engravings published in his volumes were later produced.

**Fig. 10:**
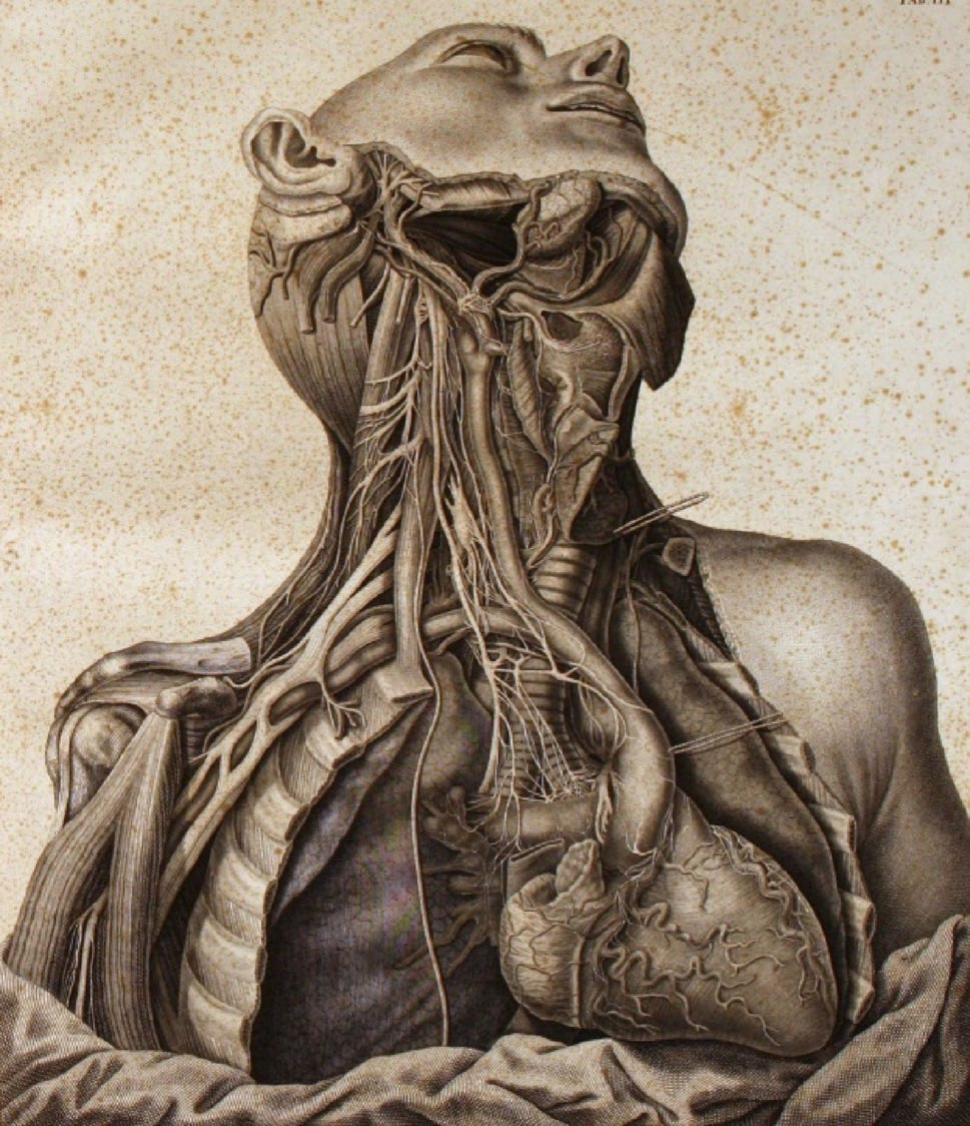
One of the illustrations of the Antonio Scarpa Tabulae [31]. Museum for the History of the University of Pavia. The details of the facial nerve, vagus nerve, cervical plexus from which the phrenic nerve descends, and the brachial plexus are fascinating

One of his most remarkable works is the *Tabulae neurologicae ad illustrandam historiam Anatomicam cardiacorum nervorum*,* noni nervorum cerebri*,* glossopharyngaei*,* et pharyngaei ex octavo cerebri*, published in Pavia in 1794 by the printer Baldassarre Comino [31]. This publication was the result of years of research conducted by Scarpa on the nervous system. It is a large volume containing seven copper-engraved plates (five devoted to human anatomy and two to comparative anatomy), engraved by Faustino Anderloni (1766–1847)—a young artist from Brescia who later became a professor of drawing at the University of Pavia. The engravings were based on drawings executed by Scarpa himself. The volume provides a detailed description of the course of the glossopharyngeal nerve, the vagus nerve, and the hypoglossal nerve, as well as the innervation of the heart, demonstrating that the terminal branches of individual nerves are directly connected to the cardiac muscle fibres (Fig. 10).

Each plate is accompanied by a line drawing with an explanatory legend to help clarify the image.

Scarpa’s work fully belongs to the history of anatomical illustration between the eighteenth and nineteenth centuries, alongside that of other scientists such as the Scottish John Bell (1763–1820), Charles Bell (1774–1842) and John Lizars (1787–1860), who worked with his brother William, the Germans Samuel Thomas von Soemmerring (1755–1830) and Friedrich Tiedemann (1781–1861), the French Jules Germain Cloquet (1790–1883) and Jean-Baptiste Sarlandière (1787–1838), the Italian Paolo Mascagni (1755–1815) [42].

Even before representing it, Scarpa was able to perceive the beauty of the structure of the human body beyond death. In the Anatomical Museum of Pavia, specimens were prepared with particular care for their aesthetic appearance as well, featuring shaped wooden supports decorated with gold. Alongside natural preparations, Scarpa also acquired for his collection two wax statues—a man and a woman depicted life-size and partially dismountable—created in Florence by Clemente Susini (1754–1814). These are two splendid works of art that combine beauty with an accurate three-dimensional representation of the human body [15].

According to Scarpa, wax models were especially useful for representing parts for which the preservation of natural specimens was difficult; for parts whose overall structure was hard to depict from a single point of view; and finally for parts that could be observed in nature only with a microscope or that, because of their small size, could not be observed simultaneously by several people [43]. All the tools available for teaching and research at the time (anatomical plates, preparations, and models) were thus integrated with the opportunity to attend autopsies within the setting of the anatomical theatre (Table 1).

**Table 1:** Comparative summary of the three anatomical theatres

City (Venue)	Design type	Levels/seating	Illumination	Project date	Opening date	Siting within a complex
Padua (Palazzo Bo)	Permanent wooden funnel-shaped amphitheatre	~ 6 standing rings	Artificial light; daylight after 1845	1594	1595–1596	Integrated in main university building
Modena (Sant’Agostino Hospital)	Octagonal tower; later irregular hexagon	5 tiers	Daylit via openings	1773	23 Jan 1775	Hospital complex near the dissection rooms
Pavia (Cortile dei Medici e Artisti, now Cortile dei Caduti)	Semi‑amphitheatre	4 levels + gallery	Oculus + windows	1785	1785–1786	Central academic building

## Reflection on the modern legacy of anatomical theatres

Today, the historical anatomical theatres of Padua, Modena and Pavia function primarily as preserved cultural and architectural monuments, occasionally used for academic ceremonies or symbolic events. Although no longer employed for routine dissection, their pedagogical principles, visibility, direct observation, spatial hierarchy, carefully controlled lighting, and proximity to anatomical collections have been fully absorbed into modern dissection rooms and surgical skills laboratories. In this sense, Scarpa’s vision survives not only as historical memory but as a living educational framework embedded in contemporary anatomical practice.

**Fig. 11:**
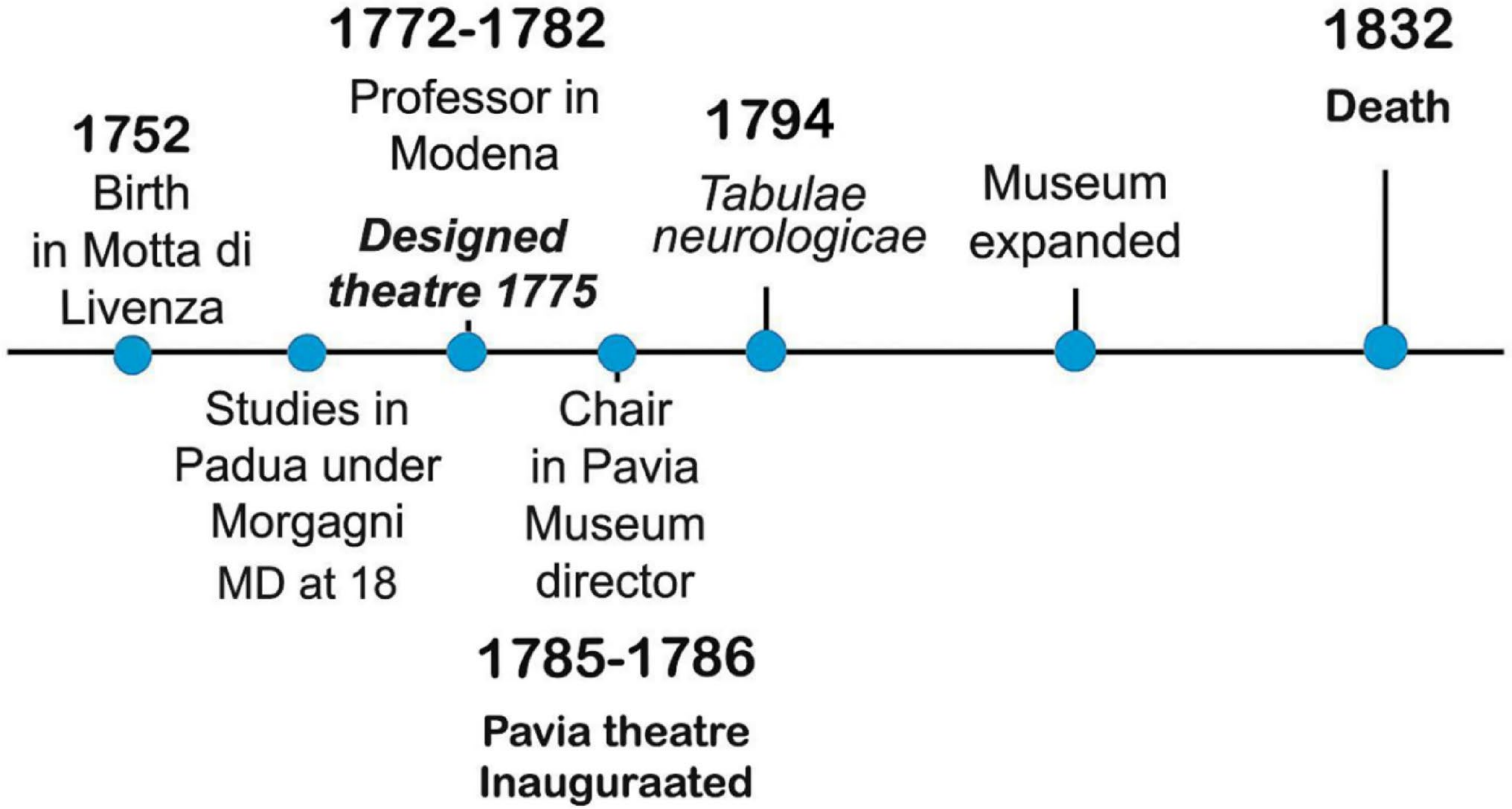
Chronology summarising the main moments of Scarpa’s life and scientific roles, his involvement with specific theatres and anatomical museums

## Conclusions

The study highlights Antonio Scarpa’s dedication to teaching facilities (Fig. 11), exemplified by his consideration of the connection between clinical anatomy and the places where it had to be taught not only in the most scientifically valid way, but also in the most comfortable, pleasant and artistic way. The anatomical theatre in Padua, the first of its kind, was a model for many European anatomists due to its functionality and design, and it was the place where Scarpa was born as a scientist and acquired his educational ideals. Scarpa valued teaching as crucial for anatomical science progress, focusing on the ideal study of spaces, lighting, and aesthetics. Scarpa, influenced by his experiences in various anatomical theatres, consistently aimed to replicate the Padua theatre’s success in his teaching spaces, but adding very modern details, embryos of current dissecting theatres. Scarpa’s influence extended beyond anatomy to the humanities, highlighting the educational and cultural significance of the theatres, for example, wanting to associate them with anatomical museums. This fact underlines how much care of the workplace, the architectural aspects, and suitability to the needs of students, anatomists, architects, and surgeons made Scarpa also a pioneer of multidisciplinarity in anatomical science.

## Data Availability

No datasets were generated or analysed during the current study.
